# Intrajejunal Infusion of Levodopa-Carbidopa Gel Can Continuously Reduce the Severity of Dropped Head in Parkinson’s Disease

**DOI:** 10.3389/fneur.2017.00547

**Published:** 2017-10-16

**Authors:** Hiroshi Kataoka, Yasuhiko Sawada, Tadashi Namizaki, Naotaka Shimozato, Hitoshi Yoshiji, Satoshi Ueno

**Affiliations:** ^1^Department of Neurology, Nara Medical University, Kashihara, Japan; ^2^Third Department of Internal Medicine, Nara Medical University, Kashihara, Japan

**Keywords:** Parkinson, levodopa-carbidopa intestinal gel, dropped head, antecollis, intrajejunal infusion

## Abstract

Dropped head can occur in patients with Parkinson’s disease and make their quality of life unpleasant because they cannot obtain a frontal view. The pathophysiologic involvement of dopamine agonist or central or peripheral mechanisms has been proposed. Levodopa therapy with the withdrawal of dopamine agonists was sometimes effective, but the effect in most patients did not persist for the entire day. We describe a patient with Parkinson’s disease whose dropped head responded throughout the day to the continuous intrajejunal infusion of levodopa-carbidopa intestinal gel (LCIG). During off-periods before treatment with LCIG, severe akinesia and freezing of gait were evident, and she could not continuously obtain a frontal view because of the dropped head. About 20 min after the intrajejunal infusion of LCIG, these features remarkably improved, and she could obtain a frontal view. The angle of dropped head was improved from 39.39 to 14.04°. This case suggests that infusion of LCIG can reduce the severity of dropped head for a longer period than oral levodopa.

## Introduction

Dropped head can occur in patients with Parkinson’s disease and make their quality of life unpleasant because they cannot obtain a frontal view. The pathophysiologic involvement of dopamine agonist or central or peripheral mechanisms has been proposed (1). Levodopa therapy with the withdrawal of dopamine agonists was sometimes effective, but the effect in most patients did not persist for the entire day. We describe a patient with Parkinson’s disease whose dropped head responded throughout the day to the continuous intrajejunal infusion of levodopa-carbidopa intestinal gel (LCIG).

## Case Report

**Figure 1:**
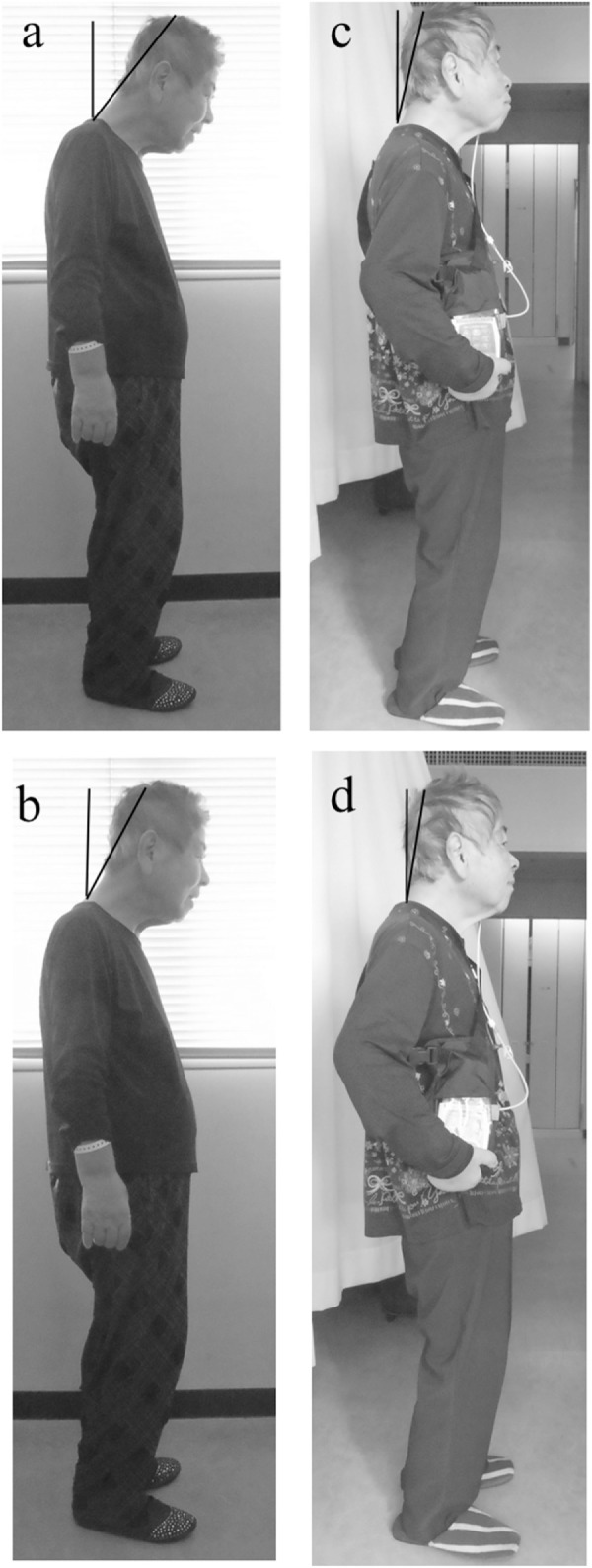
A patient with dropped head before **(A,B)** and after **(C,D)** continuous intrajejunal infusion of levodopa-carbidopa intestinal gel (LCIG). After infusion of LCIG, the angle of antecollis was reduced in a natural position **(A–C)** and in a position with the neck averted with maximum effort **(B–D)**. The black line is located between the vertex (Cz) and seventh spinous process.

Eleven years ago, a 72-year-old right-handed woman noticed left-hand tremor. In 2006, she consulted a hospital physician, and cogwheel rigidity, resting tremor in the right hand, and akinesia were present. These features responded to treatment with oral levodopa (200 mg/day). In February 2008, she received levodopa (200 mg/day) and pramipexole (0.5 mg/day). She showed signs of moderate parkinsonism, including resting tremor in the right hand, right-dominant rigidity in all four limbs, and akinesia. In August 2009, the dose of pramipexole was increased to 1 mg/day because of the resting tremor. She complained that her trunk became bent, and lumbar pain was evident. She showed mild-to-moderate trunk flexion. Pramipexole was discontinued, and the symptoms resolved. Since 2010, the severity of akinesia increased, and gait difficulty gradually developed. In June 2014, she received levodopa (300 mg/day), selegiline (10 mg/day), and zonisamide (50 mg/day). In May 2015, wearing-off was evident, and the dose of levodopa was increased to 400 mg/day. In October, peak-dose dyskinesia was evident, and zonisamide was withdrawn. In April 2016, wearing off and troublesome dyskinesia developed. She also noticed painful lower abdominal contractions several times during off-periods in the night. Treatment with zonisamide (25 mg/day) was therefore resumed, but the lower abdominal contractions did not resolve. In October, she noticed dropped head while receiving a stable dosage of levodopa (400 mg/day), selegiline (10 mg/day), and zonisamide (25 mg/day). Since two phenotypes of levodopa-responsiveness exist in patients with abnormal posture including dropped head (2), we performed intravenous dopamine challenge testing. The intravenous infusion of levodopa (50 mg) reduced the severity of dropped head, and the dose of levodopa was therefore increased to 500 mg/day. The severity of dropped head mildly decreased. In early November, the dose of zonisamide was increased (50 mg/day) because of prolonged off-periods, and subsequently rotigotine (9 mg/day) was begun. In the middle of November, dropped head redeveloped. Rotigotine was discontinued, and the dose of levodopa was increased to 600 mg/day (in 3 divided doses per day) while continuing stable doses of selegiline (10 mg/day) and zonisamide (50 mg/day). Dropped head and wearing-off were unchanged, and dyskinesia further developed. She was admitted to our hospital in December. The frequency of levodopa treatment was increased to five times per day. The dyskinesia mildly decreased, but off-periods in the morning, evening, and night were unchanged. Painful lower abdominal contractions also occurred from the evening to night. The dropped head required the patient to wear a neck collar to obtain a frontal view all day. In December, she received LCIG *via* a nasoduodenal tube. She had no history of psychosis or hallucinations. The Mini-Mental Score Examination score was 26. Dropped head was evaluated by taking photographs of the lateral aspects of the head, both at rest and during the patient’s best effort to avert her neck. The angle of dropped head was calculated with the use of “Image J” software (3) by drawing a line between the vertex (Cz) and the seventh spinous process, as described previously (2) (Figure 1).

During off-periods before treatment with LCIG, severe akinesia and freezing of gait were evident, and she could not continuously obtain a frontal view because of the dropped head (Videos S1 and S2 in Supplementary Material). The severity of dropped head increased during off-periods and mildly decreased during on-periods. About 20 min after the intrajejunal infusion of LCIG, these features remarkably improved, and she could obtain a frontal view (Videos S3 and S4 in Supplementary Material). The Hoehn-Yahr stage decreased from 4 to 2, and balance also improved. The axial sum scores (items 8, 12, and 13) on the new revised Unified Parkinson’s Disease Rating Scale (UPDRS)-III (4) decreased from 9 to 1. The score on part III of the revised UPDRS decreased from 69 to 11.

The angle of dropped head was 39.39° before the infusion of LCIG (Figure 1A). On the day when the infusion of LCIG was begun, the severity of dropped head improved enough for the patient to obtain a frontal view. From day 5 after starting the LCIG infusion, the dose of LCIG (levodopa/carbidopa: 22/5.5 mg/h) was not changed. Seven days after starting LCIG, we repeatedly examined the angle of the dropped head. The angle in the natural position improved to 14.04° (Figure 1C). On maximum effort to avert the neck, the angle decreased from 28.17 to 9.73° (Figures 1B,D). This good status was maintained until the evening. After the evening on the same day, the angle deteriorated to 31.53° in the natural position and 22.62° on maximum effort to avert the neck. However, these angles were less than those before the infusion of LCIG. These serial evaluations were performed during on-periods, and the Hoehn-Yahr stage was 2 during all evaluations. The score of dyskinesia on items 1 and 2 of part IV of the newly revised UPDRS (4) decreased from 4 and 3 to 1 and 1, respectively. The patient did not complain of painful abdominal contractions. This condition was maintained for 1 day after the second evaluation. On the first night, she had transient visual hallucinations only during the infusion of LCIG (levodopa/carbidopa: 28/7 mg/h). Written informed consent was obtained from the patient for the publication of this case report.

## Discussion

Treatment with oral levodopa improves head position, but this finding was inconsistent previously (1). In our study, dropped head rapidly responded to LCIG, and this response to levodopa persisted for a prolonged time. LCIG has not been previously reported to be effective against dropped head or axial symptoms. Doherty et al. mentioned that antecollis might be an off-state phenomenon or a dyskinesia-like phenomenon related to dopaminergic medications (1). Recently, we studied patients with PD who had anterior trunk flexion, dropped head, or lateral trunk flexion and investigated whether abnormal posture responded to levodopa by means of intravenous dopamine challenge testing (2). In patients with abnormal postures related to the “off” state, the change in the angle was significantly greater than that of patients whose abnormal posture was not related to the “off” state. This observation was also seen in patients with dropped head (2). Our patient also had dyskinesia and mild fluctuations of dropped head, and the dropped head in the present patient responded to intravenous dopamine challenge testing. Intermittent intake of oral levodopa leads to fluctuating plasma levodopa levels due to erratic gastric emptying and variable jejunal absorption. This variability in plasma levodopa concentrations leads to non-physiologic intermittent or pulsatile stimulation of dopamine receptors, which can cause wearing-off or dyskinesia, especially in a dopamine-depleted state (5). As for bioavailability, LCIG results in approximately 50% lower variability in levodopa concentrations than does oral administration of levodopa-carbidopa (6). This leads to shorter off-periods or milder dyskinesia. In addition, LCIG was shown to be absorbed faster than oral levodopa in a pharmacokinetics study in patients with advanced Parkinson’s disease (6). Rapid absorption in addition to stable delivery of levodopa might be responsible for the prolonged response of dropped head to levodopa.

## Conclusion

After infusion of LCIG, dropped head in our patient deteriorated after the evening, and the ability to maintain a good status of dropped head appeared to be limited. However, this case suggests that infusion of LCIG can reduce the severity of dropped head for a longer period than oral levodopa.

## Ethics Statement

No investigations or interventions were performed outside of routine clinical care for this patient. As this is a case report, without experimental intervention into routine care, no formal research ethics approval was required. Written, fully informed consent was received from the patient. This case study reports routine clinical care provided for a patient only.

## Author Contributions

HK was responsible for the overall study design and contributed to analysis and interpretation of the data and wrote the manuscript. HK, YS, TN, and NS contributed to running the study and the acquisition of data. HK, HY, and SU contributed to drafting and critical revision of part of the submitted materials.

## Conflict of Interest Statement

The authors declare that the research was conducted in the absence of any commercial or financial relationships that could be construed as a potential conflict of interest.

## Supplementary Material

Videos S1 and S2 The severity of Parkinson’s disease before the continuous intrajejunal infusion of levodopa-carbidopa intestinal gel.

Videos S3 and S4 The severity of Parkinson’s disease after the continuous intrajejunal infusion of levodopa-carbidopa intestinal gel.
